# Characterising low-cost sensors in highly portable platforms to quantify personal exposure in diverse environments

**DOI:** 10.5194/amt-12-1-2019

**Published:** 2019-08-30

**Authors:** Lia Chatzidiakou, Anika Krause, Olalekan A. M. Popoola, Andrea Di Antonio, Mike Kellaway, Yiqun Han, Freya A. Squires, Teng Wang, Hanbin Zhang, Qi Wang, Yunfei Fan, Shiyi Chen, Min Hu, Jennifer K. Quint, Benjamin Barratt, Frank J. Kelly, Tong Zhu, Roderic L. Jones

**Affiliations:** 1 Department of Chemistry, University of Cambridge, Cambridge, CB2 1EW, UK; 2 Atmospheric Sensors Ltd, Bedfordshire, SG19 3SH, UK; 3 MRC-PHE Centre for Environment & Health, Imperial College London and King’s College London, London, W2 1PG, UK; 4 College of Environmental Sciences and Engineering, Peking University, Beijing, 100871, China; 5 Department of Analytical, Environmental and Forensic Sciences, King’s College London, London, SE1 9NH, UK; 6 Department of Chemistry, University of York, York, YO10 5DD, UK; 7 The Beijing Innovation Center for Engineering Science and Advanced Technology, Peking University, Beijing, 100871, China; 8 NIHR Health Protection Research Unit in Health Impact of Environmental Hazards, King’s College London, London, SE1 9NH, UK; 9 National Heart and Lung Institute, Imperial College London, SW3 6LR, UK

## Abstract

The inaccurate quantification of personal exposure to air pollution introduces error and bias in health estimations, severely limiting causal inference in epidemiological research worldwide. Rapid advancements in affordable, miniaturised air pollution sensor technologies offer the potential to address this limitation by capturing the high variability of personal exposure during daily life in large-scale studies with unprecedented spatial and temporal resolution. However, concerns remain regarding the suitability of novel sensing technologies for scientific and policy purposes. In this paper we characterise the performance of a portable personal air quality monitor (PAM) that integrates multiple miniaturised sensors for nitrogen oxides (NO_*x*_), carbon monoxide (CO), ozone (O_3_) and particulate matter (PM) measurements along with temperature, relative humidity, acceleration, noise and GPS sensors. Overall, the air pollution sensors showed high reproducibility (mean ${\bar{R}}^{2}$ = 0.93, min–max: 0.80–1.00) and excellent agreement with standard instrumentation (mean ${\bar{R}}^{2}$ = 0.82, min–max: 0.54–0.99) in outdoor, indoor and commuting microenvironments across seasons and different geographical settings. An important outcome of this study is that the error of the PAM is significantly smaller than the error introduced when estimating personal exposure based on sparsely distributed outdoor fixed monitoring stations. Hence, novel sensing technologies such as the ones demonstrated here can revolutionise health studies by providing highly resolved reliable exposure metrics at a large scale to investigate the underlying mechanisms of the effects of air pollution on health.

## 1 Introduction

Emerging epidemiological evidence has associated exposure to air pollution with adverse effects on every major organ system (Thurston et al., 2017). Most of this evidence comes from western Europe and North America (Newell et al., 2017) as population-scale air pollution health studies have largely relied on available outdoor air pollution measurements from fixed monitoring stations (COMEAP, 2018). Due to limitations in the availability of monitoring networks in low- and middle-income countries (LMICs), the effects of air pollution on health have been under-researched in these settings. A clear need exists for more direct epidemiological evidence in diverse geographical settings with varying air pollution sources considering the high likelihood that health effects of air pollution are not linear and cannot be simply transcribed from the western world to LMICs (Tonne, 2017).

Secondly, the low spatial and temporal resolution of exposure metrics at postcode level or coarser, which are often employed in large-scale epidemiological research, cannot separate the individual health effects of pollutants, which are generally highly correlated at these coarser scales. Additionally, outdoor measurements cannot capture the *total* personal exposure that results from the cumulative effects of an individual moving between different indoor and outdoor microenvironments. During daily life, peak exposure events often occur during commuting (Karanasiou et al., 2014) while the indoor environment is a significant site for exposure in part because people spend as much as 90 % of their time indoors (Klepeis et al., 2001). Indoor air is affected by outdoor pollutants penetrating building envelopes with additional indoor sinks, sources and emissions from building materials which cannot be detected by fixed outdoor monitoring networks. The lack of information on indoor environments at the population scale is a significant factor in poorly quantified health risks. As a result, inaccurate personal exposure estimations to air pollution introduce both bias and error in health estimations, ultimately preventing epidemiological research from moving from general to specific associations (Zeger et al., 2000).

Rapid advancements in novel sensing technologies of air pollution sensors now offer the potential to monitor detailed personal exposure during daily life at the population scale, thanks to their significantly reduced cost, smaller size and fast response. Instrument development is accelerating fast with a growing number of companies utilising combinations of such sensors (Cross et al., 2017) as well as auxiliary components to build different types of monitors (Morawska et al., 2018). As a special case, it is now estimated that there are currently over 30 000 sensors operating in China to monitor concentrations of air pollutants (Morawska et al., 2018). Several studies over the last 15 years have attempted to quantify personal exposure to air pollutants by employing portable sensors, but most of those studies have been restricted to small-scale surveys (Steinle et al., 2013). However, large-scale studies are necessary to assess the health effects of harmful pollutants because they are often seen in only small subgroups of the population due to varying individual susceptibility and exposure profiles. Novel sensing technologies are in fact the only method to expand the personal exposure coverage at the population level. Yet, concerns remain about the validation and quality control of those sensors (Castell et al., 2017) as few personal exposure studies have evaluated their performance in field deployment conditions (Rai et al., 2017). Typically, novel sensing platforms are exclusively evaluated in outdoor static co-locations with reference instruments and they only target small numbers of pollutants, most commonly ozone, nitrogen dioxide (Lin et al., 2015) and/or particulate matter (Holstius et al., 2014; Feinberg et al., 2018).

To address these shortcomings, a highly portable personal air pollution monitor (PAM) that measures a large number of chemical and physical parameters simultaneously has been developed. This paper aims to evaluate the performance of the PAM when capturing total personal exposure to air pollution in diverse environmental conditions. To do so, the PAM performance was assessed in well-characterised outdoor, indoor and commuting microenvironments across seasons and different geographical settings. The PAM has already been deployed to participants of two large cardiopulmonary cohorts in China (Han et al., 2019) (AIRLESS- Theme 3 APHH project) (Shi et al., 2019) and the UK (COPE) (Moore et al., 2016), and in a number of smaller international pilot projects in North America, Europe, South and East Asia, and Africa. This is the first of a series of publications that aim to capture total personal exposure to a large number of pollutants at unprecedented detail, and together with medical outcomes, to identify underlying mechanisms of specific air pollutants on health. As the field of novel air pollution sensing technologies expands rapidly, this paper further aims to provide methodological guidance to researchers from diverse disciplines on how to comprehensively calibrate and validate portable monitors suitable for personal exposure quantification.

## 2 The personal air quality monitor

**Figure 1:**
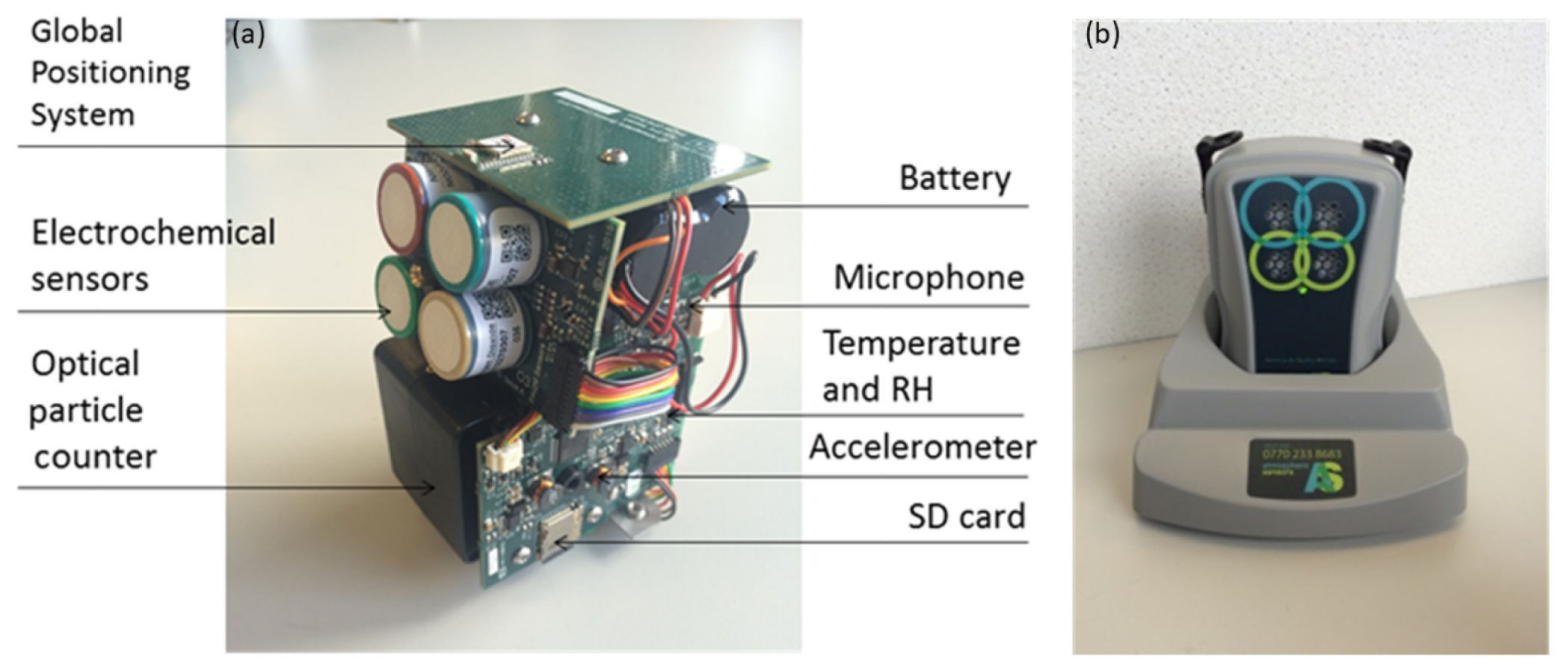
The personal air quality monitor. **(a)** Design of the PAM platform internals and **(b)** PAM charging inside the base station. The external dimensions of the PAM are 13 cm × 9 cm × 10 cm.

The PAM has been developed at the Department of Chemistry, University of Cambridge in collaboration with Atmospheric Sensors Ltd. It is now commercially available (independently from the University of Cambridge) from Atmospheric Sensors Ltd (model AS520, http://www.atmosphericsensors.com, last access: 22 August 2019). The PAM (Fig. 1) is an autonomous platform that incorporates multiple sensors of physical and chemical parameters (Table 1). The compact and lightweight design of the PAM (ca. 400 g) makes the unit suitable for personal exposure assessment. The PAM is almost completely silent and can operate continuously. No other input is required by the user other than to place it for periodic charging (e.g. daily) and data upload in a base station. The measurements are also stored in an SD card inside the monitor and uploaded through a general packet radio service (GPRS) to a secure access FTP server. Customised system software has been developed to optimise the performance of the platform. Depending on the chosen sampling interval of either 20 s or 1 min, the battery life on a single charge lasts for 10 h or 20 h respectively. The combined cost of the sensors alone is less than GBP 600 and the total cost of the PAM is less than GBP 2000, making it a “lower-cost” system (Cross et al., 2017).

User-friendly, bespoke software (Fig. S1) has been developed to automate the management and post-processing of the large volume of raw data collected with the PAM network. Data are held in a PostgreSQL relational database management system, which has an unlimited row-storage capacity and allows the querying of large quantities of data in a flexible manner while maintaining performance as the volume of data grows. Post-processing was performed in R software (R Development Core Team, 2008) (Fig. S1) following the methodology outlined in this paper.

### 2.1 Measurements of CO, NO, NO_2_ and O_3_

The principle of operation of all commercially available miniaturised gaseous sensors currently involves measuring changes in specific properties of a sensing material (e.g. electrical conductivity, capacitance, mass, optical absorption) when exposed to a gas species (Morawska et al., 2018). The PAM integrates small (20 mm diameter) electrochemical (EC) sensors based on an amperometric principle of operation (Stetter and Li, 2008) for the quantification of carbon monoxide (CO), nitric oxide (NO), nitrogen dioxide (NO_2_) and ozone (O_3_). These EC sensors are the A4 variant from Alphasense (NO-A4, Alphasense Ltd, 2016a; CO-A4, Alphasense Ltd, 2017a; NO_2_-A43F, Alphasense Ltd, 2016b; Ox-A431, Alphasense Ltd, 2017b) and operate on a fourelectrode system. The principle of operation of the four-electrode system is identical to that of the earlier variants of the three-electrode system (Alphasense, 2013a) where the conventional setup of working electrode, counter electrode and reference electrode is supplemented with an additional electrode, the auxiliary (or non-sensing) electrode, to compensate for the temperature dependence of the cell potential (Popoola et al., 2016). Earlier variants of EC sensors used in this paper have been extensively characterised in laboratory conditions and in static outdoor dense sensor networks (Mead et al., 2013). Those studies provided evidence that, after appropriate post-processing, the sensors had a linear response to the targeted pollutants and achieved excellent performance with limits of detection (LOD) < 4 ppb demonstrating their suitability for atmospheric air quality measurements. The linearity and LOD of the four-electrode sensors (when integrated in the PAM) have been tested under laboratory conditions following the same methodology as described in Mead et al. (2013) yielding very similar results.

Currently, standards for the calibration and performance evaluation of EC sensors focus on industrial applications (British Standards Institution, 2017). Following those standards, a widely adopted approach to calibrate EC sensors is gas chamber experiments to determine offset (baseline) and sensitivity (gain). To address the lack of standards for novel sensing technologies, a number of researchers and governmental organisations are developing protocols and guidelines to evaluate sensor and monitor performance in the laboratory and in the field, such as the European Metrology Research Programme of EURAMET (Spinelle et al., 2013), the European Standardisation Committee (CEN/TC 264/WG 42, 2018) and US-based groups (Long et al., 2014; AQ-Spec, 2017).

Building on those protocols, the EC sensors were calibrated by co-location with certified reference instruments in similar environmental conditions and the same geographical area where the monitors had been or were to be deployed. The considerable advantage of this approach over laboratory calibration includes the exposure of the sensor to the actual air pollution and temperature–relative humidity conditions under which it is expected to operate, as well as the assessment of any site-specific potential cross-interferences. A linear regression model (Eq. 1) was applied to the co-location data to determine the calibration parameters used to convert raw sensor signals (mV) to mixing ratios (ppb). Temperature effects were corrected through the auxiliary electrode (AE), which might have a different sensitivity to the working electrode (WE, *a* ≠ *b*). The cross-sensitivities between the NO_2_ and O_3_ measurements were corrected via parameter *c* (the cross-sensitive gas *Y* is NO_2_ for O_3_ measurements and vice versa). As the CO and NO sensors were found to be sufficiently selective, *c* was set to zero for the calibration of those sensors.

(1) $${\left[X\right]}_{\text{ref}}=a{\text{WE}}_{X}+b{\text{AE}}_{X}+c{\text{WE}}_{Y}+d,$$

where [*X*]_ref_ is the reference measurement of pollutant *X* (ppb), *a* is the sensitivity of the working electrode (ppb mV^−1^), WE_*X*_ and AE_*X*_ are the raw signal of the working and auxiliary electrodes respectively (mV), *b* is the sensitivity of the auxiliary electrode (ppb mV^−1^) (accounts for temperature), *c* is the cross sensitivity with gas *Y* (ppb mV^−1^; *c* = 0 for CO and NO), WE_*Y*_ is the raw signal of the working electrode of the cross-sensitive gas *Y* (mV), and *d* is the intercept (ppb).

To evaluate the performance of the linear model, the datasets were split into training (i.e. calibration) and validation periods to first extract the calibration parameters and then apply them to the validation set and compare the measurements with those from reference instruments (referred to as the “calibration–validation” method). The training sets ranged from 1 to 16 d, and the adjusted coefficient of determination $\left({\bar{R}}^{2}\right)$ remained stable for training periods longer than 3 d. Therefore, approximately a third of the dataset was selected as a training set. As relationships in these linear models should ideally not be extrapolated beyond the range of the observations (including meteorological conditions), the calibration periods covered the temperature and concentration ranges in which the sensors were deployed (Cross et al., 2017). Once the performance of the model was established in diverse environments, we used the full co-location periods to determine the agreement between PAM sensors and reference instruments.

### 2.2 Particulate mass measurements

The operation of virtually all miniaturised particulate matter (PM) sensors that are currently commercially available is based on the light-scattering principle, either volume scattering devices or optical particle counters (OPCs) (Morawska et al., 2018). The PAM integrates a commercially available miniaturised OPC (Alphasense OPC-N2; Alphasense Ltd, 2018), which uses Mie scattering for real-time aerosol characterisation (Mie, 1908). Particles pass through a sampling volume illuminated by a light source (in this case a laser) and scatter light into a photodetector (Bohren and Huffman, 1983). The amplitudes of the detected scattering signal pulses are then related to particle size. The OPC counts these pulses and typically sorts them into different particle size bins (Walser et al., 2017). The OPC-N2 classifies particles in 16 sizes (bins) in the range 0.38–17 μm. The performance of this OPC in the laboratory (AQ-Spec, 2017; Sousan et al., 2016) showed a high degree of linearity. Similarly studies evaluating the OPC performance in outdoor static deployments (Di Antonio et al., 2018; Crilley et al., 2018) showed that once site- and season-specific calibrations were applied, the miniaturised sensor could be used to quantify number and mass concentrations of particles with a precision similar to other standard commercial reference optical PM instruments.

The complexity of evaluating PM sensor performance is much greater than that of gas sensors. Compared with standard instrumentation, optical PM instruments face four inherent limitations which introduce potential differences in mass estimations compared with reference gravimetric methods.

- Exposure of the particles to relative humidity (RH) results in hygroscopic growth of particles and leads to mass overestimation (Di Antonio et al., 2018).
- Small variations in the sensitivities of the photodetector and the intensity/angle of the laser may result in a systematic error specific to each OPC sensor. Additionally, as particles enter the optical chamber, they may deposit on internal surfaces and optics of the sensor, leading to a reduction in the measured scattered light and thus instrument sensitivity.
- A further limitation of all optical methods is their inability to detect particles with diameters below a certain size, typically 200–400 nm (Morawska and Salthammer, 2003).
- Finally, optical methods cannot distinguish the physical and chemical parameters of the aerosol (e.g. density, hygroscopicity, volatility), which might vary significantly as people move between different microenvironments with diverse emission sources, further increasing the uncertainty of mass estimation.

(a) To compensate for these limitations, this work first corrected for the effect of RH by applying an algorithm based on the particle size distribution which was developed for aerosols in urban environments (Di Antonio et al., 2018). (b) In the second step, a scaling factor for each OPC was determined to account for sensor–sensor variability. This scaling factor was determined from a linear fit between the RH-corrected mass and the reference measurements for each season independently and to compensate for instrument sensitivity that may change over time. (c) As the reference instruments (e.g. TEOM) include particles below the size range of the OPC in their mass estimations, the scaling factor partly addresses the under-prediction of mass due to undetected smaller particles which may vary between seasons. The varying aerosol composition (d) remains a challenge, and therefore a constant density of 1.65 g cm^−3^ was assumed. Although the OPC is able to measure PM_1_, PM_2.5_ and PM_10_, this paper focusses on the performance of the PM_2.5_ measurements because of the availability of reference instruments.

## 3 Performance of the PAM under well-characterised conditions in the field

In the following three sections the performance of the PAM is assessed when measuring air pollution concentrations in different environments that are relevant for the quantification of total personal exposure (outdoor, indoor and in movement) in the UK and China. Sensor performance may vary significantly with season (e.g. temperature and RH artefacts) while meteorological conditions may affect the variation in outdoor air pollution levels directly (e.g. stability of the atmosphere) and indirectly by socioeconomic patterns (e.g. increased energy demand for heating). Similarly, indoor air may be directly affected by outdoor air pollution levels and indirectly through occupants’ behavioural patterns (e.g. window adjustment to achieve thermal comfort). Taking into account the strong seasonal variation in air pollution levels, the performance of the PAM was evaluated by co-locating one or multiple PAMs with reference instruments during both the “heating” (when the majority of householders heat their home on a regular basis) and “non-heating” seasons. The residential central heating season in Beijing is from 15 November to 15 March (Beijing municipal government), while in the UK the equivalent heating season is 5.6 months (October–March/April) (BRE, 2013).

**Figure 2:**
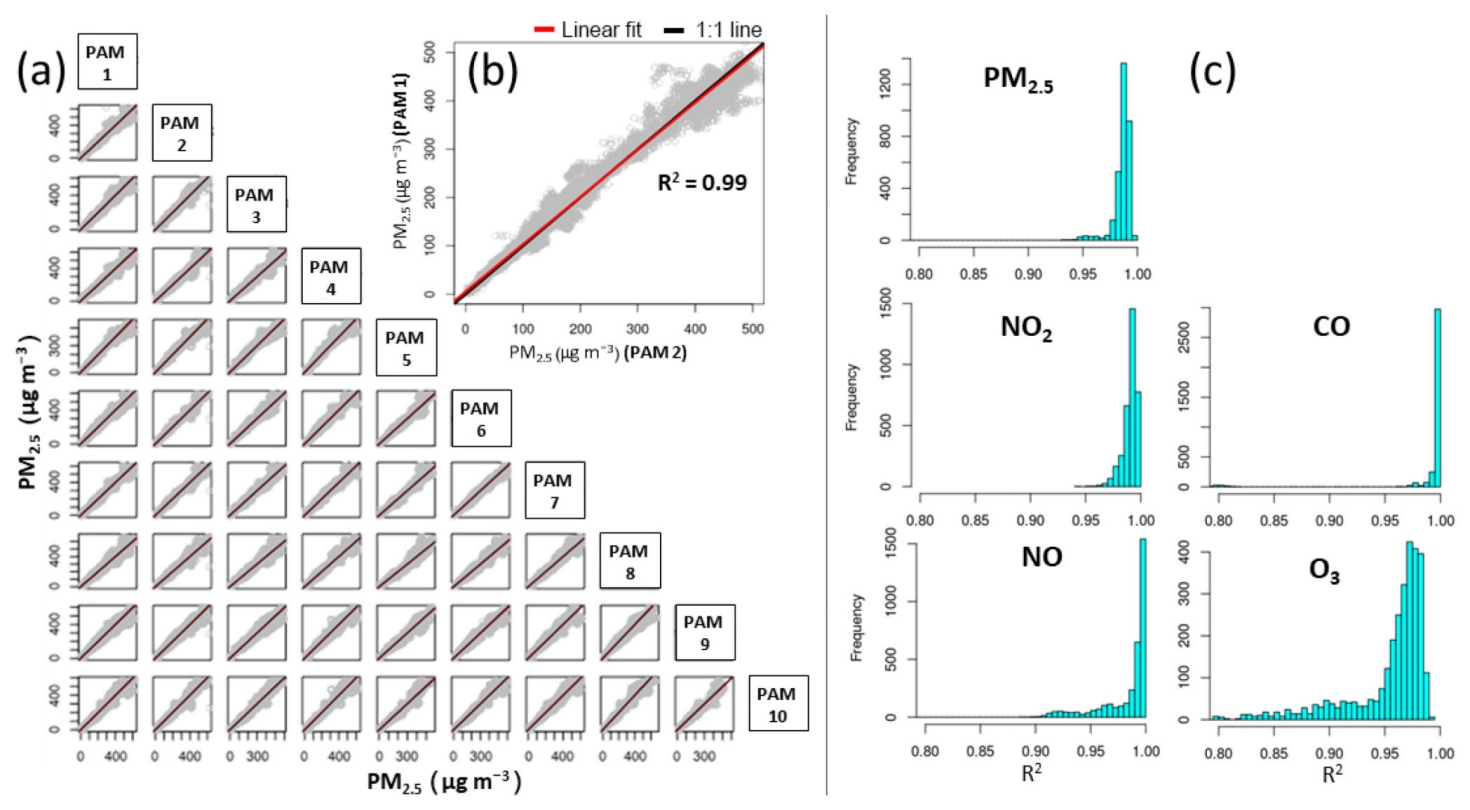
Reproducibility of a PAM network (in that case 60 monitors) co-located outdoors in Beijing during the heating season after 1 month of field deployment. **(a)** Scatterplot of the PM_2.5_ measurements between 10 sensor pairs. The 1 : 1 line is in black, and the linear fit line is in red. **(b)** Close-up of a scatterplot from **(a)** of one representative sensor pair. **(c)** Histogram of the coefficient of determination (*R*^2^) between all sensor pairs. *R*^2^ values during this deployment were higher than 0.90 for all pollutants indicating the high reproducibility of the sensors’ readings (see Table S1 for all co-locations). O_3_ sensors *R*^2^ > 0.80 due to very low ambient levels close to the LOD of the sensors.

The description of the sites, principle of operation and models of certified reference instrumentation used can be found in Table 2. The co-locations in China involved 60 PAMs which had been previously deployed to 250 participants of a cardiopulmonary cohort for 1 month during the heating season and 1 month during the non-heating season (Han et al., 2019). The co-location in the UK involved 60 PAMs that have been previously deployed to 150 participants of a COPD cohort for 2 years continuously (Moore et al., 2016). The reproducibility between co-located sensors was very high even when the ambient concentrations were close to the LOD (mean *R*^2^ ≥ 0.80 for EC sensors and *R*^2^ ≥ 0.91 for the OPC; see Fig. 2 and Table S1 in the Supplement). Hence, the performance of the selected PAMs in static deployments as described in this section is representative and can be extrapolated to the entire sensor network.

### 3.1 Outdoor performance of sensors in diverse urban environments with varying pollution profiles and meteorological parameters

In total, four outdoor co-location deployments have been evaluated to comprehensively characterise the performance of the sensors (in the UK and China during the heating and non-heating seasons; see Table 3). The PAMs were placed in protective shelters close to the inlets of the certified air pollution monitoring stations. The sensor measurements were converted to physical units following the methodology described in Sect. 2.1 and 2.2.

**Figure 3:**
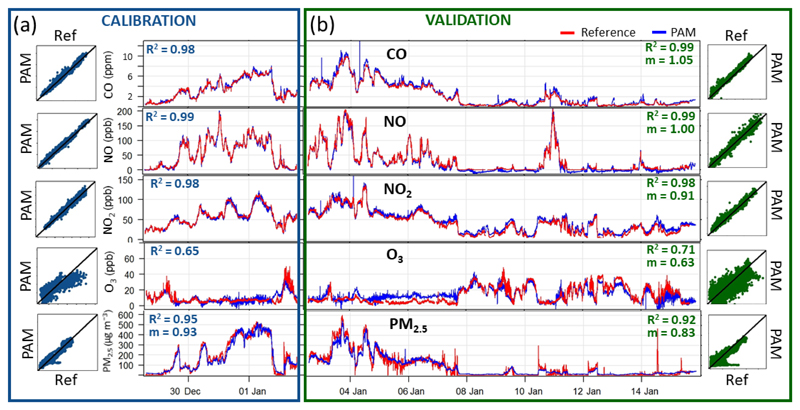
Outdoor co-location of one representative PAM with calibrated reference instruments in China (winter 2016/2017) at 1 min time resolution demonstrating the calibration–validation methodology to evaluate the performance of the linear model. The first 5 d **(a)** were used to calibrate the EC sensors. The remaining co-location data (14 d, **b**) were used to validate the extracted calibration parameters. The scatterplots on each side show the correlations between reference and PAM measurements with the 1 : 1 line in black ${\bar{R}}^{2}$ and gradients (*m*) are shown on each side in the corresponding colour.

As an illustrative example, the outdoor co-location in Beijing, China (19 days, December 2016 to January 2017), is presented in Fig. 3 to demonstrate the previously mentioned calibration–validation method (Sect. 2.1). The time series of the pollutants measured by the PAM (blue) closely follow the reference instruments (red) in both the calibration (Fig. 3a) and validation (Fig. 3b) periods. Similarly, the time series and scatterplots of the other three co-locations (UK in the heating season, China and UK in the non-heating season) can be found in the Supplement (Figs. S2–S4).

Table 3 gives a quantitative overview of the agreement between the PAM measurements and the reference instruments in outdoor co-locations during the heating and non-heating seasons. Ambient temperature and RH (median, range: 5 %–95 %) as well as the mean and maximum pollutant concentration measured are presented to describe the ambient conditions of each co-location. Because the PAM internal temperature is on average 7 °C higher than the ambient temperature due to heat generated by the internal battery, the internal conditions the sensors were exposed to are also presented. The sensor performance was evaluated against the reference instruments using (1) ${\bar{R}}^{2}$ of the linear regression between PAM and the reference and (2) the root-mean-square error (RMSE) using both the validation and calibration periods (Table 3). ${\bar{R}}^{2}$ may be a misleading indicator of sensor performance when measurements are taken close to the LOD of the instruments. The RMSE can be a complementary parameter of ${\bar{R}}^{2}$ for the evaluation of performance, as it summarises the mean difference between measurements from the sensor and certified instruments. The average values of ${\bar{R}}^{2}$ and RMSE of all N sensors during all co-locations are given in Table 3.

#### 3.1.1 Outdoor performance of the PAM during the heating season co-locations

During the heating season outdoor co-locations of a number of PAMs next to certified reference instruments, ambient temperatures ranged from −4 to 6 °C in China and between 4 and 14 °C in the UK. Air pollution in China was characterised by elevated levels of CO and PM_2.5_ (Table 3) for extended time periods (haze events) partially driven by stagnant winds or a weak southerly wind circulation (Shi et al., 2019). Compared with pollutant levels in the UK, the concentrations of CO and PM_2.5_ were approximately 10 times higher while the contrast in ambient NO_2_ levels was less marked with levels in China only approximately 3-fold higher.

The O_3_, NO and NO_2_ sensors exhibited an excellent performance (${\bar{R}}^{2}$ ≥ 0.84) in both geographical settings (Table 3). The median RMSE values were close to the LOD of the sensors (< 3 ppb) in the UK and slightly higher in China (< 12 ppb) (Fig. 3, Table 3). In both deployments, the RMSE values of these gaseous sensors were negligible compared to the ambient concentration ranges of the targeted pollutants (less than 16% of the maximum mixing ratio recorded by the reference instruments).While the median ${\bar{R}}^{2}$ between the CO sensor and the corresponding reference was reasonably high in both outdoor deployments (≥ 0.74), the median RMSE values were also quite large (< 32 ppb). In fact, this is due to the known high intrinsic noise and LOD of the reference instrumentation (> 40 ppb, Thermo Fischer Scientific, 2017), which is much higher compared to that of the electrochemical sensors (LOD < 4 ppb; see Sect. 2.1).

Following the correction of the size-segregated particle measurements for the effect of RH (Sect. 2.2), the PM mass quantification with the miniaturised OPC agrees with the TEOM reference instrument with an adjusted ${\bar{R}}^{2}$ of 0.93. The low RMSE values (> 8.6% of the maximum concentration) demonstrate that the scaling factor adequately addresses the under-prediction of mass due to undetected smaller particles when derived from field calibration in the local environment. Due to unavailable measurements, the PM measurements in the UK could not be corrected for RH effects, which resulted in only a moderate correlation with the reference instrument (${\bar{R}}^{2}$ = 0.57, Fig. S2).

#### 3.1.2 Outdoor performance of the PAM during the non-heating season co-locations

One outdoor co-location in China (Fig. S3) and one in the UK (Fig. S4) were performed during the non-heating season, both over periods of 2 weeks (Table 3). In the UK, seasonal variation in ambient temperatures, RH and pollution levels was relatively small. In contrast, in China, seasonal variation was large with ambient temperatures reaching up to 36.3 °C (median: 29.9 °C) and generally lower pollution levels compared to the heating season. However, in both geographical settings, O_3_ was significantly elevated. The performance of the O_3_ sensor remained reliable in all deployments with median ${\bar{R}}^{2}$ = 0.80 and RMSE values < 15 ppb, which might provide valuable insights into the health effects of this pollutant because (a) ozone is a strong oxidant with a high potential to affect the body (Nuvolone et al., 2018) and (b) has the highest concentrations during the non-heating season compared to other pollutants which usually peak during the heating season.

Due to a malfunction of the PM reference (TEOM) instrument during the non-heating season at PKU, the PAM PM measurements had to be compared with a TEOM installed at a nearby governmental site (Haidianwanliu). Although not closely co-located (~ 3 km), the gradient between the PAMs and reference measurements was close to unity (average *m* = 0.96, see example Fig. S3) and there was still a notable correlation (${\bar{R}}^{2}$ = 0.65) with a median RMSE of 25 μg m^−3^, indicating that away from direct sources PM concentrations are essentially homogenous over relatively large urban areas. Compared with the heating season, PM concentrations in China were significantly lower, whereas PM levels in the UK varied little with season. After correcting for the effects of RH on PM, the PAM performance in the UK during the non-heating season significantly improved compared with the heating season (RMSE = 2 μg m^−3^ within the particle size range 0.38–17 μm).

While the performance of the O_3_ and OPC sensors remained reliable across seasons and geographical settings, the performance of the CO, NO and NO_2_ sensors decreased significantly (${\bar{R}}^{2}$ ≥ 0.20) during the hottest parts of the non-heating season in China due to extreme temperatures (internal median temperatures of the PAM: 40.2 °C, 5 %–95 %: 32.7–45.8 °C, Table 3). It should be noted that NO levels were close to the LOD of the sensor, which also affects the ${\bar{R}}^{2}$ values. We conclude that the measurements of the CO, NO and NO_2_ sensors should be interpreted with caution when the sensors are exposed to temperatures above 40 °C. However, during the field deployment to participants, the sensors were exposed to lower temperatures (see Fig. S5) that did not impact on their performance (see Sect. 3.2).

### 3.2 Indoor performance of the NO_2_ and PM sensors

Low-cost air pollution sensors have generally been characterised outdoors next to reference instruments as described in the previous section. However, little is known about the performance of these sensors in indoor environments, where people spend most of their time (Klepeis et al., 2001), and environmental conditions (e.g. temperature, RH) and emission sources may be significantly different compared with nearby outdoor environments.

To evaluate the indoor performance of the NO_2_ and the OPC sensors, an experiment in an urban flat in central Beijing was performed during the non-heating season (May 2017). One PAM was deployed in the living area next to two commercial instruments that were used to provide reference measurements: (1) a cavity attenuated phase shift spectroscopy instrument (CAPS Teledyne T500U) for NO_2_ and (2) a portable commercial spectrometer (GRIMM 1.108) for particulate matter measurements (Table 2). During the experiment the occupants relied on natural ventilation, adjusting the windows freely to achieve thermal comfort. Median indoor temperatures were 26.0 °C (5 %–95 % range: 17.1–28.8 °C), and the median internal PAM temperature was 33.0 °C (5 %–95 % range: 24.3–36.2 °C), which is comparable with the temperature range during the non-heating season field deployment to participants (internal median temperature: 35.0 °C, 5 %–95% range: 28.5–39.9 °C, Fig. S5).

**Figure 4:**
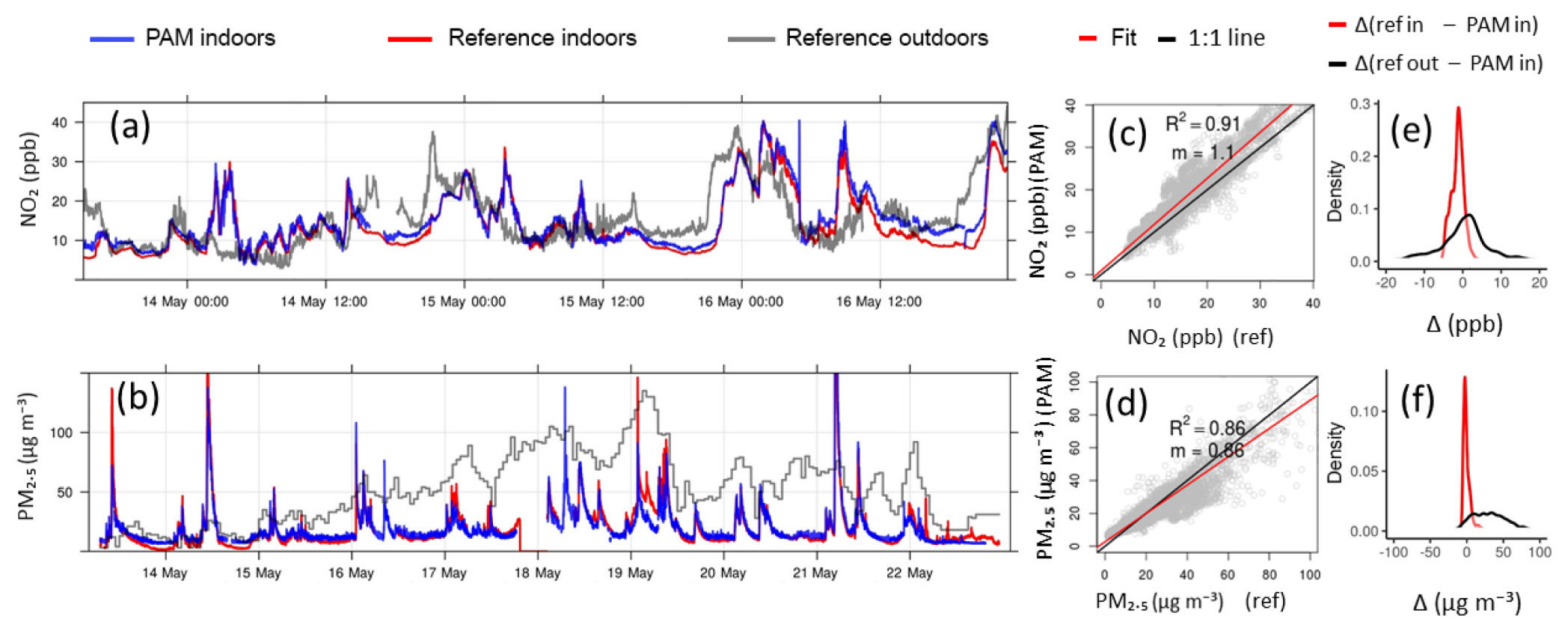
Indoor co-location of a PAM with portable commercial instrumentation (Table 2) in an urban flat in China during the non-heating season. **(a)** Time series of NO_2_ from the PAM (blue) and a cavity attenuated phase shift spectroscopy (CAPS) instrument (red). Outdoor NO_2_ measurements (grey) were collected at a PKU reference site (Table 2), which was located 5.3 km away. Time resolution of measurements is 1 min. **(b)** Time series of PM_2.5_ mass measured with the PAM (blue) next to a commercial portable spectrometer (GRIMM 1.108, red). Mass concentrations were calculated from particle counts within the size range 0.38–17 μm with the same aerosol density for both instruments. Outdoor PM_2.5_ mass measurements (grey) were collected at the closest governmental station (Table 2, 1 h time resolution), which was located 6 km away. **(c, d)** Scatterplots show an excellent agreement between commercial instruments and miniaturised sensors, making them suitable for the quantification of indoor pollution levels. The 1 : 1 line is in black and gradient *m* in red. **(e, f)** Density plots of the difference between measurements from the PAM and the indoor reference (red) are compared with the difference between the PAM and the outdoor reference (black).

The conversion of the raw measurements to parts per billion used the sensitivities extracted using outdoor co-locations during both the heating and non-heating seasons (Sect. 3.1) with the linear model (Sect. 2.1). The performance of the low-cost sensors in the indoor environment (Figs. 4 and S6) was comparable to the outdoor performance demonstrated in the previous section (${\bar{R}}^{2}$ = 0.91, gradient *m* = 1.1, RMSE = 3 ppb for NO_2_ (Fig. 4c) and ${\bar{R}}^{2}$ = 0.86, gradient *m* = 0.86, RMSE = 7 μg m^−3^ for PM_2.5_ (Fig. 4d)), proving their suitability to quantify indoor air pollution levels for these species provided they have been adequately calibrated in the local environment.

Although this short experiment is only a “snapshot” of indoor exposure, it shows that the measurement error of the PAM relative to established commercial instruments is negligible compared with the error in indoor exposure estimates introduced from using inadequate exposure metrics, in this case outdoor measurements from the closest monitoring reference site. For example, using outdoor measurements from the closest monitoring station would have resulted in an over-prediction of indoor PM_2.5_ concentrations (moderated by attenuation effects of the building envelope) with an average difference of 30 μg m^−3^ (standard deviation: 29 μg m^−3^), which is significantly higher than the 7 μg m^−3^ RMSE value of the PAM (Fig. 4f). While indoor NO_2_ levels broadly followed outdoor levels, the range of the error in under-predicting and over-predicting exposure events is much broader (min–max range: −18 to 18 ppb; Fig. 4e) compared with the error introduced from measurement uncertainties (−7 to 5 ppb). Such peak exposure events might be important triggers for acute health responses.

### 3.3 Performance of the PAM in non-static configurations

The aim of this section is to evaluate the PAM reproducibility and accuracy while in movement, with pedestrian and invehicle deployments.

#### 3.3.1 Reproducibility of the PAM when not static

**Figure 5:**
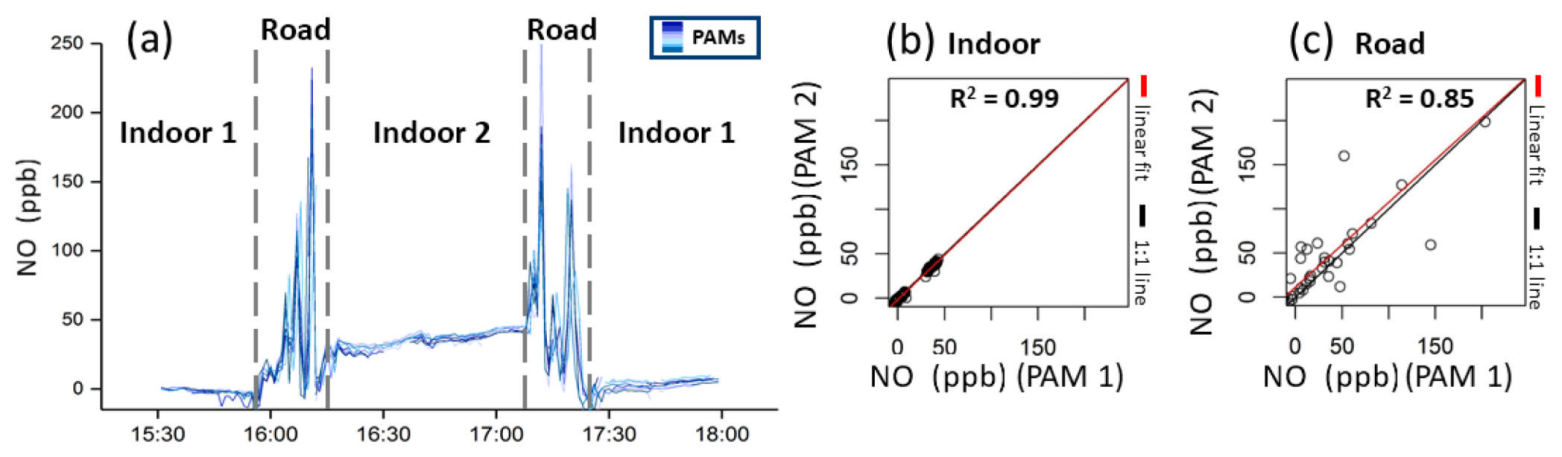
Short-term deployment of nine PAMs carried simultaneously by a pedestrian moving between two indoor environments (laboratory, café) in Cambridge, UK, in January 2018. **(a)** Time series of NO measurements from the PAM sensors (blue lines). **(b, c)** Scatterplots between two of those PAMs, whereby indoor data were separated from outdoor data. The 1 : 1 line is in black, and the linear fit line is in red.

Multiple (in this case nine) PAMs were carried by a pedestrian while keeping an activity diary and walking between two indoor environments via a highly trafficked road in Cambridge, UK (weekday in January). Using NO measurements (the main traceable component from combustion engines) as an illustrative example, Fig. 5a shows the simultaneous measurements of all PAMs as a time series and the scatterplots between the measurements of two of those PAMs separated into indoor (Fig. 5b) and outdoor data (Fig. 5c).

Significant changes of the pollution levels were observed when moving between the different environments, illustrating the high granularity of personal exposure in daily life. Compared with the indoor environments, walking in traffic resulted in elevated pollution exposure events. As illustrated in the time series of Fig. 5, the difference in pollution levels between the three micro-environments was significantly higher than the variability between PAM measurements.

Table 4 gives an overview of the correlations within the co-located moving network. In indoor environments an excellent agreement between all sensors (median *R*^2^ > 0.96) was found, indicating a high sensor reproducibility. An exception was the O_3_ sensor, which showed poor between-sensor reproducibility due to very low indoor and outdoor concentrations (< 5 ppb) near the LOD of the sensor. The between-sensor correlations in the road environment were lower than indoors (median *R*^2^ > 0.85) due to highly heterogeneous air pollution concentrations driven by complex factors (e.g. canyon air mixing, moving vehicle sources, topology). This signifies that in such environments air pollution concentrations might differ on such short spatial and temporal scales that even sensors that are less than 1m apart from each other capture a slightly different exposure profile.

When moving rapidly between different environments with different temperatures (i.e. from outdoors to a warmer indoor microenvironment) false peaks were observed in the EC sensor measurements (Fig. S7) (Alphasense Ltd, 2013b). The response and recovery time following rapid temperature transitions was found to vary for different sensor types. To account for the false sensor responses, first an algorithm to identify those events was developed and then a 15 min window for CO and a 5 min window for NO, NO_2_ and O_3_ measurements was removed from the data (Figs. S7 and S8). Though it potentially excludes peak exposure events as rapid temperature changes often occur when people leave heated buildings and enter (colder) traffic environments to commute, this correction method typically removes less than 0.1% of the exposure dataset under daily life conditions. The PM measurements are not affected by these temperature transitions.

### 3.4 Accuracy of the PAM when not static

**Figure 6:**
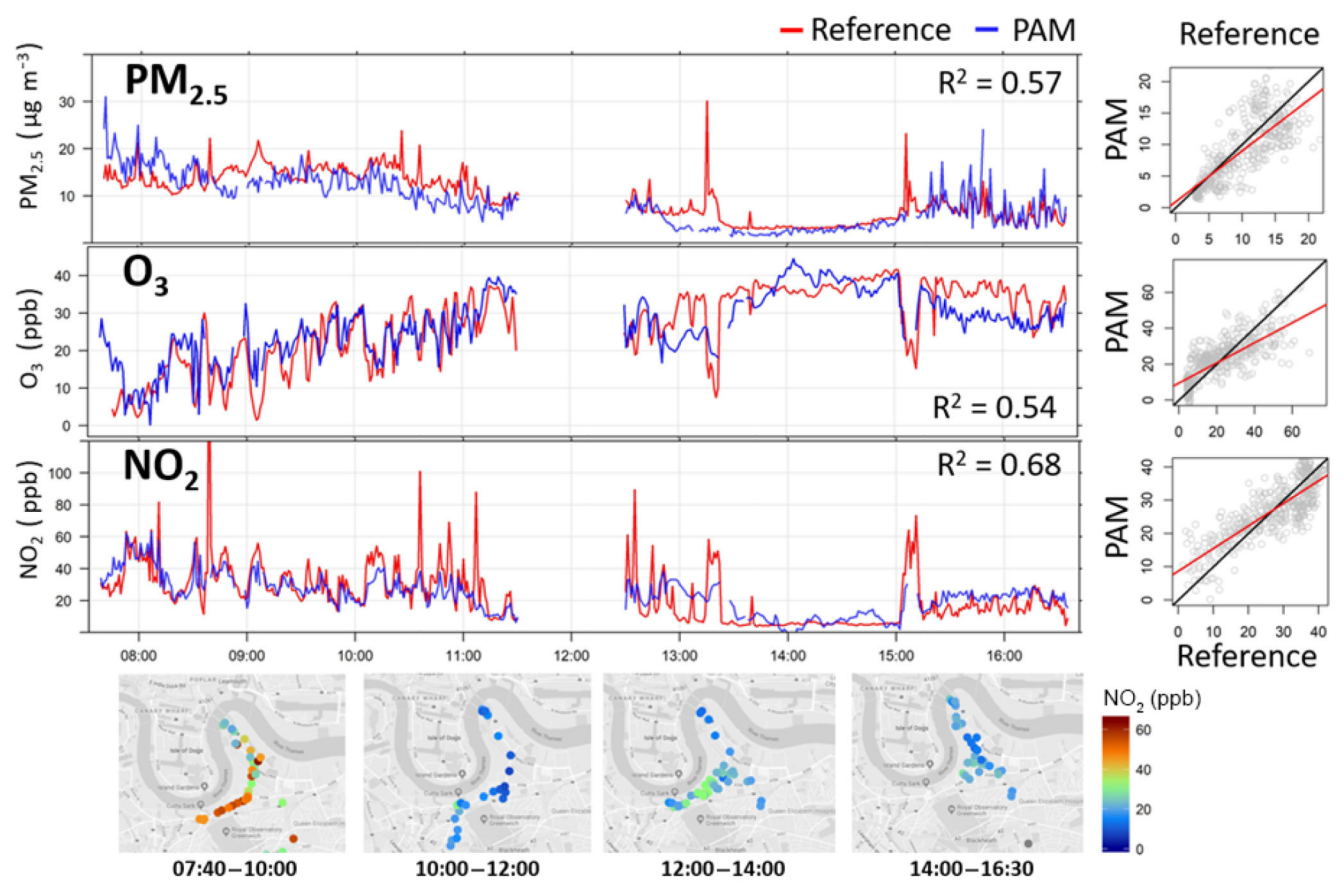
The vehicle deployment in London, UK: a PAM was attached to a car equipped with multiple commercial instruments (Table 2) for 1 d. **(a)** Time series of 1 d measurements of the PAM (blue) and commercial instruments (red). **(b)** Corresponding scatterplots between measurements from commercial instruments and the PAM in motion in an urban environment. The 1 : 1 line is in black, and the linear fit line is in red. **(c)** Maps (map data © Google 2019) of the mobile deployment over 2 h windows illustrating the large temporal variability of NO_2_.

A PAM was mounted on the roof of a battery-powered vehicle equipped with multiple commercial instruments (Table 2) mapping air pollution levels in London at speeds of up to 60 km h^−1^ for 1 d during the non-heating season (Fig. 6). The PAM was mounted on the roof with the OPC inlet facing forwards and the EC sensors facing to the sides. The reference instrument inlets were located on the car roof as well. There was no correlation between car speed and RMSE values in the gaseous and particulate measurements. The OPC contains an airflow measurement unit which compensates for any wind or internal flow dependence.

Considering the high spatial variability of air pollution in traffic environments (see Sect. 3.3.1), the accuracy of the PAM in a mobile configuration was high for all targeted pollutants $\left({\bar{R}}^{2}\geq 0.54\right)$. To illustrate the large degree of variability of air pollution concentrations over time, the investigated area was mapped throughout the day multiple times with the highest concentrations of PM_2.5_ and NO_2_ recorded during the morning rush hour.

## 4 Discussion and conclusions

Mounting evidence points towards a causal link between exposure to air pollution and health outcomes. However, due to current limitations in cost, maintenance and availability of instrumentation, most large-scale health studies have focused on developed countries and have relied on low-spatial- and low-temporal-resolution (generally outdoor) air quality data as metrics of exposure, severely limiting causal inferences in epidemiological research worldwide. Emerging low-cost sensing technologies can offer a potential paradigm shift in capturing personal exposure of the population during daily life in addressing this critical shortcoming.

In this paper we demonstrated that, with suitable calibration and post-processing, the performance of currently available low-cost air quality sensors, in this case incorporated into a highly portable personal monitor (the PAM), is comparable with the performance of reference instrumentation across a wide range of conditions:

– in diverse outdoor environments (urban background and traffic);
– across seasons (over a wide temperature and RH range);
– in two geographical settings with differing air pollution levels and meteorological profiles (UK and China);
– in indoor environments (residential, laboratory, café)
– with varying emission sources, and
– in static and in non-static deployments.

A critical important outcome of this study is that the performance of the sensors substantially exceeds that needed to quantify the differences between indoor and outdoor pollution levels, and thus to quantify exposure levels in a reliable manner.

There are certain performance caveats with the low-cost sensors used in this study, which once identified are likely to be addressed in future generations of sensors.

– The performances of the CO, NO and NO_2_ sensors were found to degrade at temperatures above 40 °C. In fact, such extreme environmental conditions were not encountered during the actual personal exposure sample periods for which the PAMs were used, and the performance criteria discussed above were met.
– A limitation of all optical PM sensors, low-cost or reference, is that they cannot measure small particles below a critical size threshold (typically 200–400 nm). In this work we show that by appropriate local calibration, this shortcoming can be largely accounted for.

The toxicity of particles is also likely to depend on their chemical composition (Kelly and Fussell, 2015). Most national networks measure total mass only, and measuring particle chemical composition is currently largely the domain of the research community. A major challenge will be to develop techniques to allow routine PM composition measurements, for both the regulatory networks and applications such as personal monitoring.

The key conclusion is that when suitably operated, highly portable air pollution personal monitors can deliver traceable high-quality exposure metrics which can address scientific, health and policy questions for the indoor and outdoor environment in a way that has not been possible before. Mobile and static PAM networks have now been deployed in a range of health studies, and these will be the focus of future papers.

## Supplementary Material

The supplement related to this article is available online at: https://doi.org/10.5194/amt-12-1-2019-supplement.

Supplementary Material

## Figures and Tables

**Table 1:** Summary of monitored parameters of the PAM. PM_1_, PM_2.5_ and PM_10_ are the fraction of particles with an aerodynamic diameter smaller than 1, 2.5 and 10 μm respectively. CO: carbon monoxide; NO: nitric oxide; NO_2_: nitrogen dioxide; O_3_: ozone.

Parameter	Method	Sampling interval
Spatial coordinates	Global Positioning System (GPS)	20 s
Background noise	Microphone	100 Hz
Physical activity	Triaxial accelerometer	100 Hz
Temperature	Band-gap IC	4 s
Relative humidity (RH)	Capacitive	4 s
PM_1_, PM_2.5_, PM_10_	Optical particle counter (OPC)	20 s
CO, NO, NO_2_, O_3_	Electrochemical sensors (EC)	100 Hz

**Table 2:** Details of the reference instruments used in this study. Time resolution of all measurements was 1 min.

Deployment	Site description	NO, NO_2_	CO	PM	O_3_
Outdoor China	Urban background in Peking University (PKU) campus, Beijing	Chemiluminescence, Thermo Fisher Scientific model 42i	Nondispersive infrared, Thermo Fisher Scientific model 48i	PM_2.5_* TEOM (tapered element oscillating microbalance)	UV absorption Thermo Fisher Scientific model 49i
Outdoor UK	Urban background at the Department of Chemistry, Cambridge	Chemiluminescence, Thermo Fisher Scientific model 42i	Nondispersive infrared, Thermo Fisher Scientific model 48i	Aerosol spectrometer FIDAS PALAS 200S	UV absorption Thermo Fisher Scientific model 49i
Indoor residential China	Indoor deployment in an urban highrise Beijing flat	NO_2_ cavity attenuated phase shift spectroscopy (CAPS) Teledyne API T500U	NA	Aerosol spectrometer GRIMM 1.108	NA
Commuting environment UK	Monitoring vehicle equipped with commercial instruments driving in central London	NO_2_ CAPS Teledyne API T500U	NA	Nephelometer (scattering) Met One ES642	UV absorption Teledyne API T400

* Due to malfunctioning of the TEOM in PKU during the non-heating season, measurements from a TEOM at a nearby governmental site (Haidianwanliu, time resolution 1 h) were used. NA: not available.

**Table 3:** Overview of sensors’ performance during outdoor co-locations in China and the UK (7 to 19 days). Median values (range: 5th–95th percentiles) of the ambient temperature and relative humidity (RH), internal temperature and RH of the platform are presented. The 95th percentile of the concentration measurements of the reference over the entire co-location period is given as the maximum concentration for each pollutant. The mean adjusted coefficients $\left({\bar{R}}^{2}\right)$ and root-mean-square errors (RMSEs) indicate the agreement between the measurements of the sensors and reference instruments. The average values of all *N* sensors for each variable are given. Co-location in China in June is shown in italics as sensors were regularly exposed to temperatures higher than 40 °C where sensors do not show linear temperature responses. The sensor reproducibility for these co-locations is presented in Table S1.

		Heating season		Non-heating season	
Location		China	UK	*China*	UK
Start date–end date		28 Dec 2016–15 Jan 2017	27 Oct–13 Nov 2017	*28 Jun–16 July 2017*	26 Mar–10 Apr 2018
(total hours of co-location deployment)		(447 h)	(408 h)	(432 h)	(342 h)

Illustrative graphical example		Fig. 3	Fig. S2	Fig. S3	Fig. S4

Ambient conditions	Ambient temp. (°C)	1.1 (−3.6–6.1)	9.3 (4.3-14.4)	*29.9 (22.8–36.3)*	8.3 (4.7–18.1)

	Ambient RH (%)	40 (15–79)	81 (61–93)	*68 (43–96)*	83 (48–93)

Internal conditions of the PAM	Internal temp. (°C)	10.5 (5.3–18.0)	15.9 (11.0–20.8)	*40.2 (32.7–45.8)*	17.7 (12.2–26.8)

	Internal RH (%)	27 (14–44)	52 (39–59)	*38 (23–55)*	52 (34–60)

Number of sensors (*N*)	(–)	*N* = 59	*N* = 3	*N* = 59	*N* = 3

CO	Maximum (mean) mixing ratio (ppb)	6845 (2561)	357 (237)	*916 (575)*	276 (192)

	${\bar{R}}^{2}$	0.98	0.74	*0.71*	0.67

	RMSE in parts per billion (percentage of max)	31 (0.5 %)	31.6 (8.9 %)	*212 (23 %)*	33.3 (12.1 %)

NO	Maximum (mean) mixing ratio (ppb)	132 (38)	19 (5)	*5 (1)*	6 (2)

	${\bar{R}}^{2}$	0.94	0.89	*0.20*	0.58

	RMSE in parts per billion (percentage of max)	11.7 (8.9 %)	3.0 (15.8 %)	*13.0 (260 %)*	2.2 (36.6 %)

NO_2_	Maximum (mean) mixing ratio (ppb)	98 (42)	35 (15)	*42 (22)*	19 (10)

	${\bar{R}}^{2}$	0.84	0.90	*0.20*	0.84

	RMSE in parts per billion (percentage of max)	11.8 (12.0 %)	3.0 (8.6 %)	*13.3 (31.7 %)*	2.6 (13.7 %)

O_3_	Maximum (mean) mixing ratio (ppb)	33 (13)	30 (16)	*109 (49)*	44 (28)

	${\bar{R}}^{2}$	0.87	0.92	*0.80*	0.89

	RMSE in parts per billion (percentage of max)	3.6 (10.9 %)	2.7 (9 %)	*14.9 (13.7 %)*	4.2 (9.5 %)

PM_2.5_	Maximum (mean) conc. (μg m^−3^)	432 (114)	32 (12)	*110 (55)*	37 (3)

	${\bar{R}}^{2}$	0.93	0.57^a^	*0.65^b^*	0.80

	RMSE in microgrammes per cubic metre (percentage of max)	37 (8.6 %)	9 (28 %)^a^	*25 (22.7 %)*^b^	2 (5.4 %)

a Due to unavailable data, PM mass measurements are not corrected for RH effects.

b Comparison with governmental station ~ 3 km away.

**Table 4:** Correlations between PAM sensors. Adjusted ${\bar{R}}^{2}$ values of each sensor pair of the simultaneously carried PAMs were determined. Median *R*^2^ values of all combinations are presented in the table below. Very low O_3_ levels (< 5 ppb) resulted in poor between-sensor correlations and are given in italics.

	Median ${\bar{R}}^{2}$	
	Indoor	Outdoor
NO	0.99	0.87
NO_2_	0.96	0.94
O_3_	*0.16*	*0.46*
CO	0.99	0.95
PM_2.5_	0.99	0.85
