# Supplementary material for: Characterising low-cost sensors in highly portable platforms to quantify personal exposure in diverse environments
Source: Atmos Meas Tech. Author manuscript; Available in PMC 2019 Sep 18. (PMC6751078; doi:10.5194/amt-12-1-2019)
Supplement: Supplementary Material [file EMS84312-supplement-Supplementary_Material.pdf]

Supplement of Atmos. Meas. Tech., 12, 4643–4657, 2019
https://doi.org/10.5194/amt-12-4643-2019-supplement
© Author(s) 2019. This work is distributed under
the Creative Commons Attribution 4.0 License.

Atmospheric Measurement Techniques
Open Access

Supplement of

Characterising low-cost sensors in highly portable platforms to quantify personal exposure in diverse environments

Lia Chatzidiakou et al.

Correspondence to: Lia Chatzidiakou (ec571@cam.ac.uk)

The copyright of individual parts of the supplement might differ from the CC BY 4.0 License.

Supplementary material

S1. Data processing with a bespoke automated R package

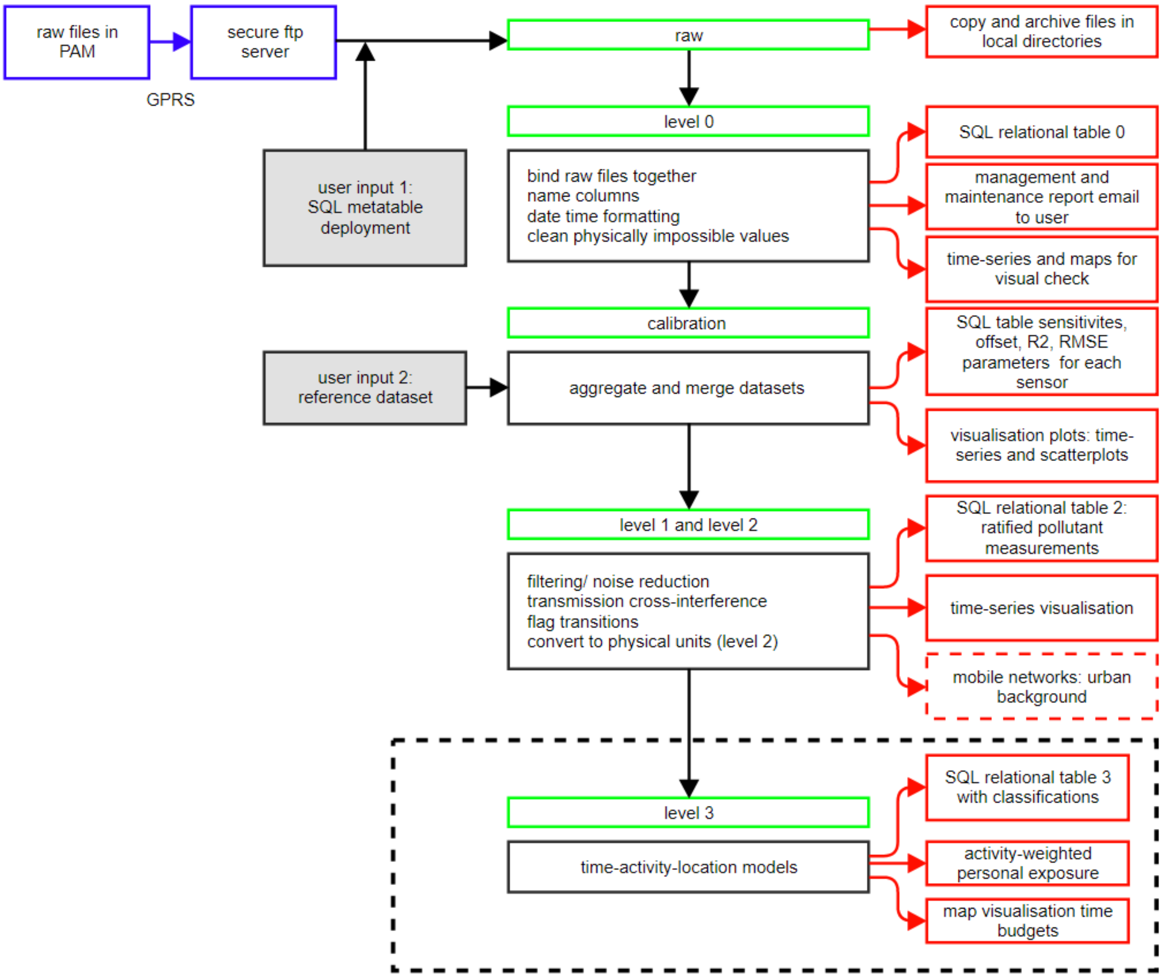

Figure S1. Data architecture of the bespoke software for the transparent and reproducible management and post-processing of data transmitted from the PAM network. Data is held in a relational database management system (RDBMS) in PostgreSQL to provide complex query capabilities and to create relationships between different components of the data itself. Data querying and post-processing is performed in R software and was selected as an established, open-source system where several independent but interoperable packages were used to address a wide range of statistical analysis and visualisation techniques. The user is required to input the metadata (grey) in the relational database containing information on deployment periods, reference instrumentation, hardware settings, maintenance schedule etc. The code can be executed with automated scheduled tasks (green) to output ratified databases (red). Level 0 produces time-series graphs and maps to facilitate fast visual inspection of the data and sends reports by email to improve efficiency of the maintenance of the PAM network. Levels 1 and 2 focus on the conversion of raw measurements to physical units and the quality assurance/ quality control of the personal exposure measurements. Dashed lines indicate work that will be presented in upcoming publications: 1) the creation of automated algorithms to classify time-activity-location profiles of individuals to estimate activity-weighted exposure; and, 2) the disaggregation of outdoor and indoor sources to overall personal exposure using information extracted from the wireless sensor network.

S2. Sensor reproducibility

To evaluate the reproducibility of the sensors, the coefficient of determination between the raw PM measurements and working electrode readings of all EC sensor pairs during the four co-locations in the UK and China in both seasons are presented in Table T1. All EC sensors show excellent correlations across the network with mean R^2 > 0.80 with the exception of the NO_2 sensors during the non-heating season in China, possibly affected by the high temperatures that the sensors were exposed to (see subsection). The coefficient of determination between the OPC-N2 sensors was high across seasons and settings ($R^2 > 0.91$); however, the gradient was lowest in the UK co-location during the heating season.

Table S1. Summary of reproducibility between sensors in outdoor co-locations in China and the UK that ranged from 7 to 19 days during the heating and non-heating season. Linear fitting equations between sensors generated the coefficient of determination (R^2) and the gradient [m] matrices. The average values of all N sensors for each variable are given, the standard deviations (σ) indicate the variation within the sensor network. The corresponding summary of the linear models with the reference instruments is presented in Table 3.

		**Heating season**		**Non-heating season**	
		China (Dec- Jan)	UK (Oct- Nov)	*China (June)*	UK (April)
	Internal PAM temperature (C)	10.5 (5.3-18.0)	15.9 (11.0–20.8)	*40.2 (32.7–45.8)*	17.7 (12.2–26.8)
	Internal RH (%)	27 (14-44)	52 (39 -59)	*38 (23 – 55)*	52 (34 – 60)
**N sensors**		N =59	N=3	*N=59*	N= 3
**CO**	Maximum mixing ratio (ppb)	6845	357	*916*	276
	R^2 (σ)	1.00 (0.01)	0.98 (0.00)	*0.94 (0.03)*	0.94 (0.00)
**NO**	Maximum mixing ratio (ppb)	132	19	*5*	6
	R^2 (σ)	0.89 (0.17)	0.80 (0.01)	*0.81 (0.07)*	0.94 (0.005)
**NO_2**	Maximum mixing ratio (ppb)	98	35	*42*	19
	R^2 (σ)	0.97 (0.04)	0.98 (0.00)	*0.60 (0.12)*	0.89 (0.03)
**O_3**	Maximum mixing ratio (ppb)	33	30	*109*	44
	R^2 (σ)	0.91 (0.05)	0.80 (0.00)	*0.90 (0.03)*	0.96 (0.01)
**$PM_{2.5}$**	Maximum conc. (μg m^{-3})	432	32	*110*	37
	R^2 (σ)	0.95 (0.17)	0.91 (0.05)	*0.96 (0.01)*	0.98 (0.01)

S3. Outdoor co-locations

S3.1 Outdoor co-locations during the heating season

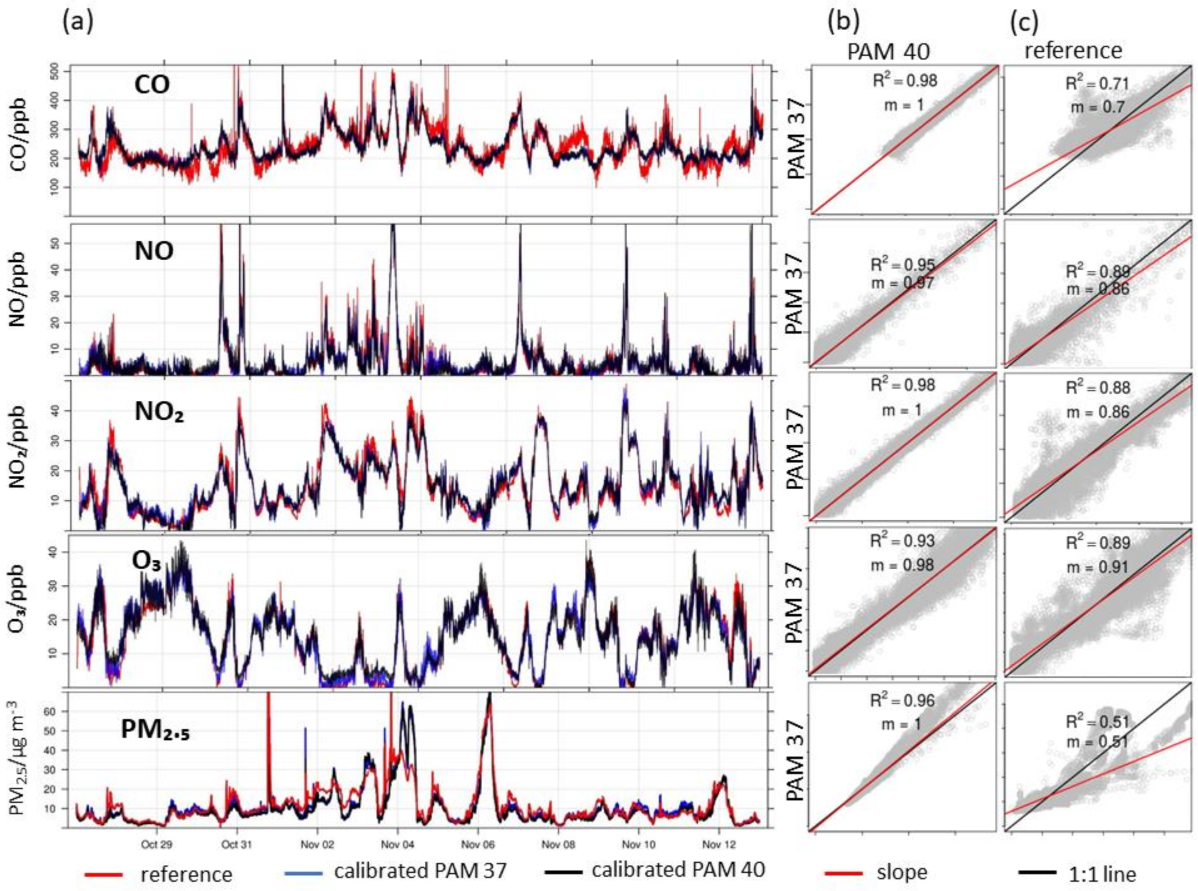

Figure S2. Outdoor co-location of PAMs with reference instruments in Cambridge for three weeks during the heating season. The PAMs had been previously deployed for two years to participants of a London cohort with chronic pulmonary disease. (a): Time series of two PAMs (blue, black) and the reference instruments (red). Time resolution is 20 sec averaged over 1 minute for comparison with the reference instruments. Due to unavailable data, the PM measurements of the PAM are presented without the RH correction resulting in an over-prediction of PM mass during certain periods. (b): correlation between PAMs and (c) correlation between PAM and reference. The 1:1 line in black; and gradients m in red.

S3.2 Outdoor co-locations during the non-heating season

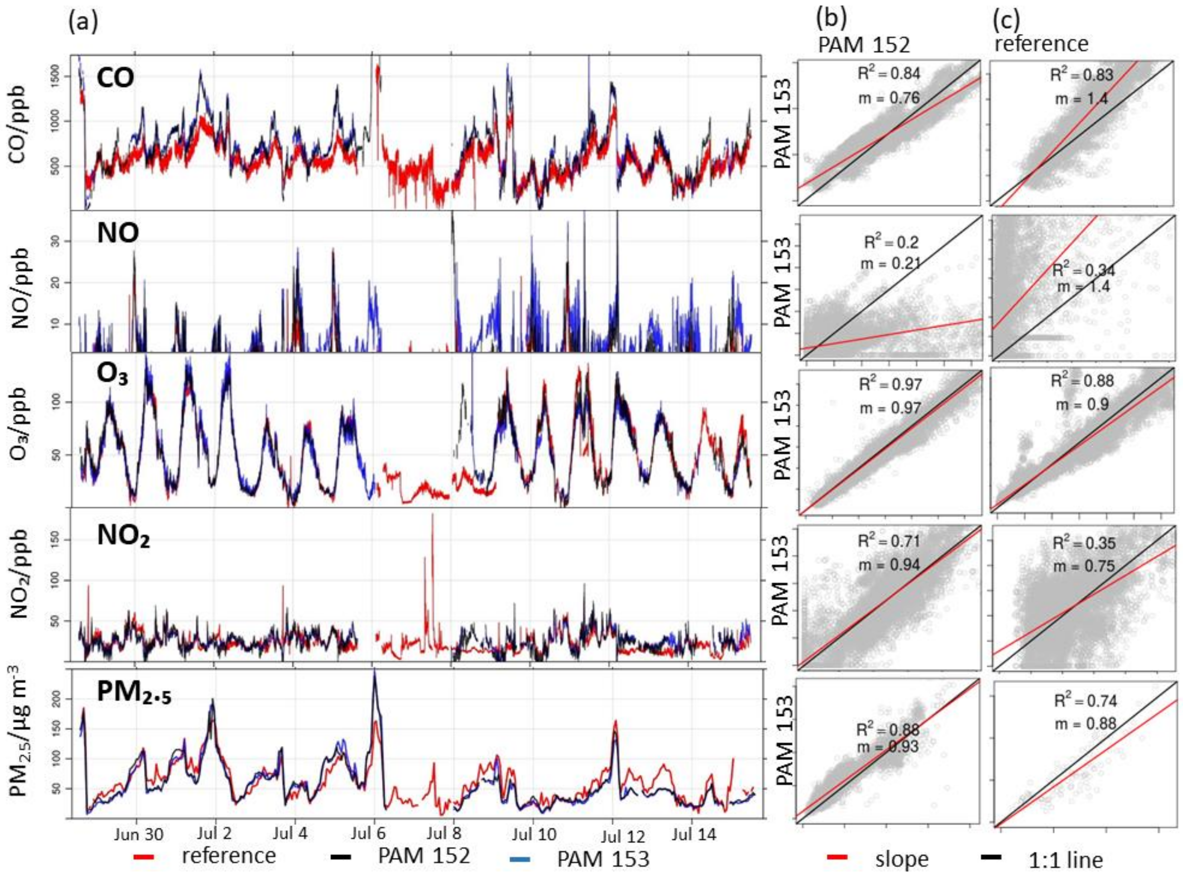

Figure S3. Outdoor co-location of PAMs with reference instruments in Beijing China during the non-heating season (June). The PAMs have been previously deployed in China for two months to participants of a cardio-pulmonary cohort. Due to malfunctioning of the TEOM in PKU during the non-heating season, measurements from a TEOM in a nearby governmental site (Haidianwanliu, time resolution 1 hour) were used for comparison with the PAM. <u>(a):</u> Time series of two PAMs (blue, black) and the reference instruments (red) in 1-minute time resolution. <u>(b):</u> correlation between PAMs and <u>(c):</u> correlation between PAM and reference. The 1:1 line (x=y) in black; and gradients m in red.

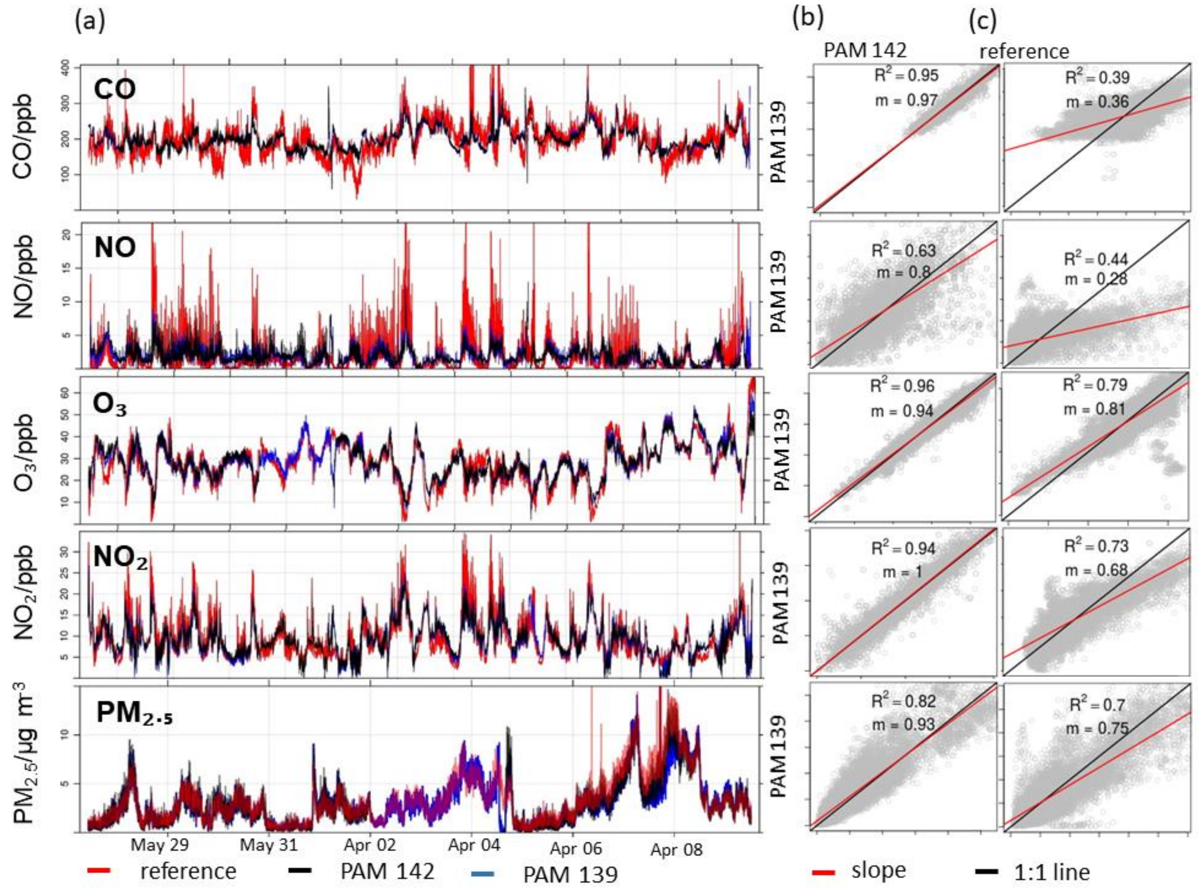

Figure S4. Outdoor co-location of PAMs with reference instruments in the UK during the non-heating season (April-May). <u>(a):</u> Time series of two PAMs (blue, black) and the reference instruments (red) in 1-minute time resolution. <u>(b):</u> correlation between PAMs and <u>(c):</u> correlation between PAM and reference. The 1:1 line (x=y) in black and gradients m in red.

S4. Deployment temperatures

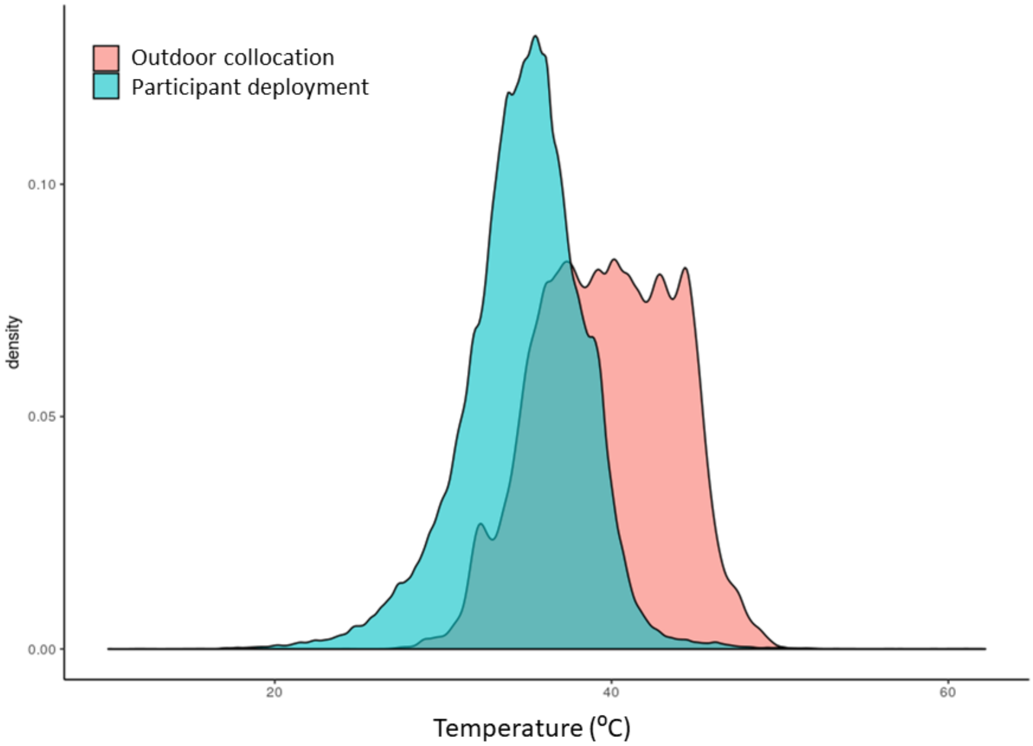

Figure S5. Histogram of internal temperatures of 60 PAMs recorded during the co-location period with reference instruments (pink) and deployment to participants (cyan) during the non-heating season in China. The co-location with the reference instruments on the roof of Peking university took place from 28/06/2017 to 16/07/2017, the field deployment was conducted in Beijing and Pinggu from 22/05/2017 to 26/06/2017. Internal temperatures of the PAMs are on average 7 °C higher than ambient temperatures.

S5. Indoor co-location

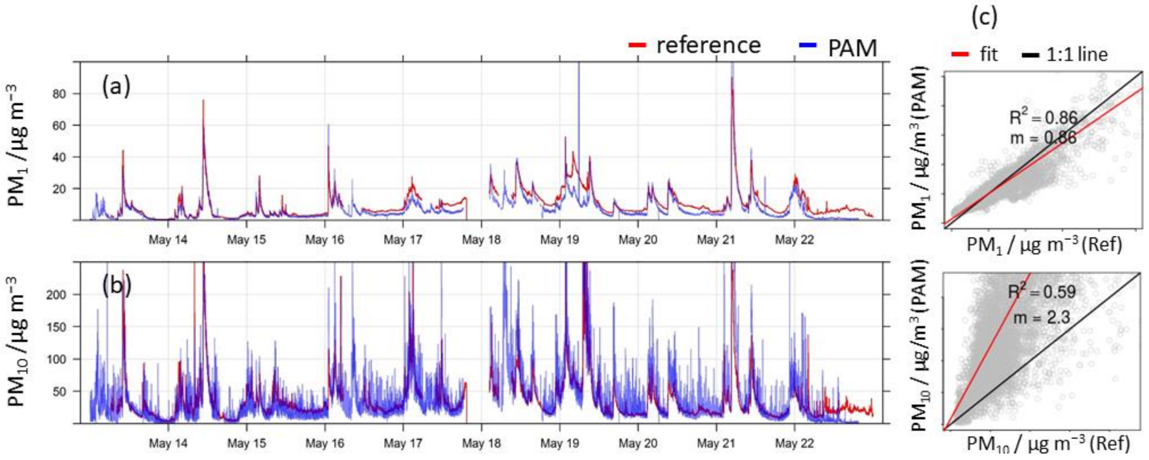

Figure S6. Indoor co-location of a PAM with portable commercial instrumentation (Table 2) in an urban flat in China during the non-heating season. Time resolution of the measurements is 1-minute. Time series of PM_1 (a) and PM_{10} (b) mass measured with the PAM (blue) next to a commercial portable spectrometer (GRIMM 1.108, red). Mass concentrations for PM were calculated from particle counts within the size range 0.38 -17 µm and same aerosol density for both instruments. <u>(c):</u> Corresponding scatterplots show an excellent agreement between commercial instrument and the PAM making them suitable for the quantification of indoor pollution levels. The 1:1 line is in black and gradient (m) in red.

S6 Effects of fast environmental changes on EC sensor performance

To investigate the effect of fast environmental changes on sensor performance, a controlled experiment was set up at a residential house in an urban background area in Cambridge. One PAM was deployed outdoor and a second PAM was deployed indoors. A third PAM was moved rapidly between an indoor and an outdoor location while a detailed time record ("diary") of the transition movements was kept (Figure S7, top). Commonly encountered residential indoor emission sources such as cooking (e.g. 11:30) and cigarette smoking (e.g. 17:30, PM measurements) were then introduced. The temperature difference between the indoor and the outdoor microenvironment was about 15°C (from 25°C indoors to 10°C outdoors) resulting in an RH difference of about 20% (30% indoors vs 50% outdoors). While the temperature sensor inside the PAM showed a relatively slow response when moved between indoors and outdoors, a rapid response of the RH sensor was evident (Figure S7, derivative of RH). The environmental changes were identified based on the time-derivative of the RH and were clearly distinguished from emission events (cooking, smoking). PM measurements were not affected by the rapid temperature changes; however, short-term transient changes were noticed in the measurements of the EC sensors. Figure S8 demonstrates that the recovery time of the NO, NO_2 and O_3 sensors was shorter (< 5 min) than that of the CO sensor (15 min).

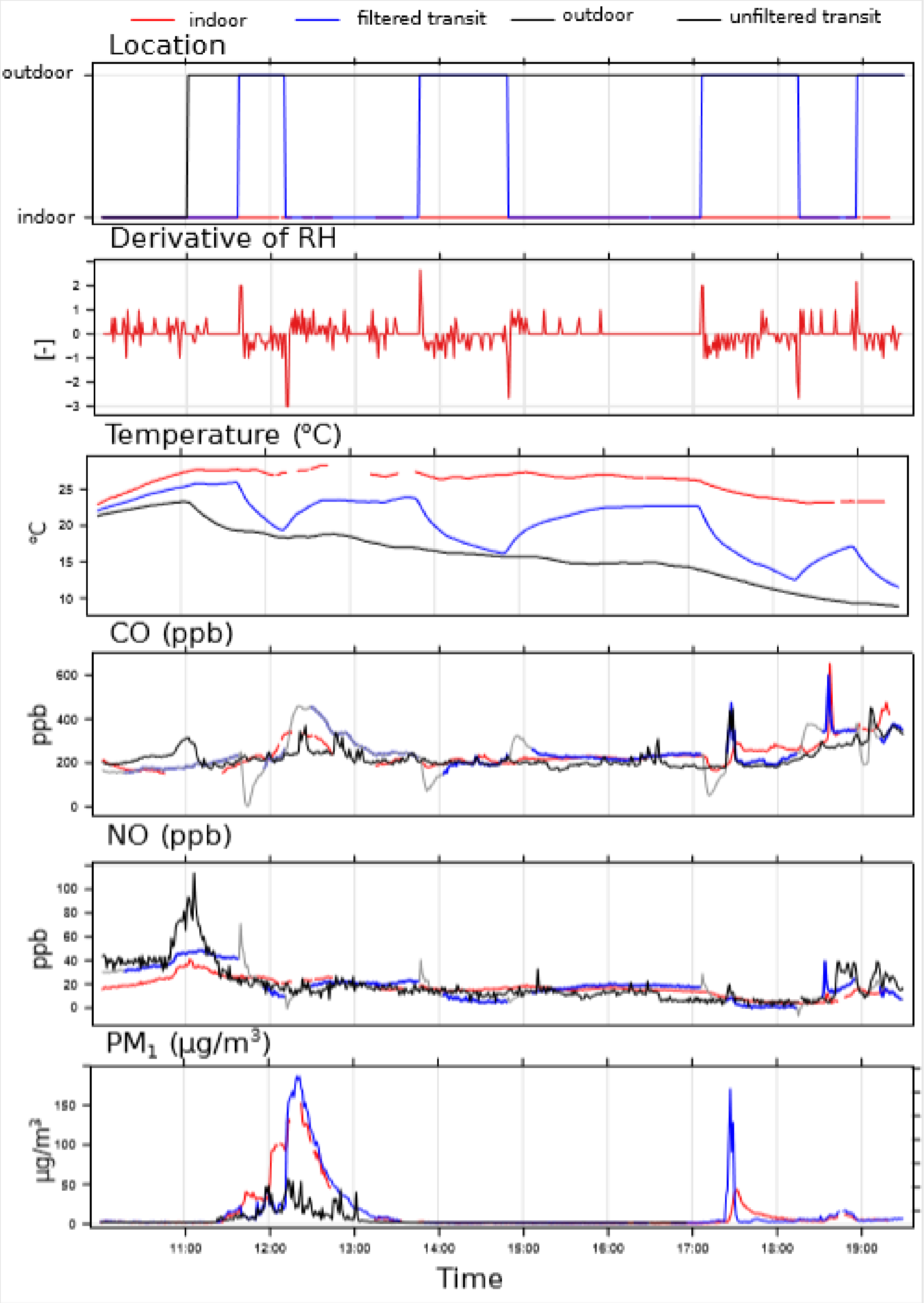

Figure S7. An experiment to characterise sensor responses to rapid environmental changes between different microenvironments. Time-series of one PAM deployed outdoors (black), a second PAM was deployed indoors (red) and a third PAM (grey, original signal) was moved rapidly between the indoor and the outdoor environment in the premises of a residence in an urban background area in Cambridge, UK. The rapid temperature changes resulted in sharp responses of the EC sensors whereas the PM measurements stay unaffected. A selective algorithm based on the on the time-derivative of the RH (dRH) was applied on the data of the moving PAM to flag and remove the false responses (blue line, filtered data) without filtering out short-term pollution events.

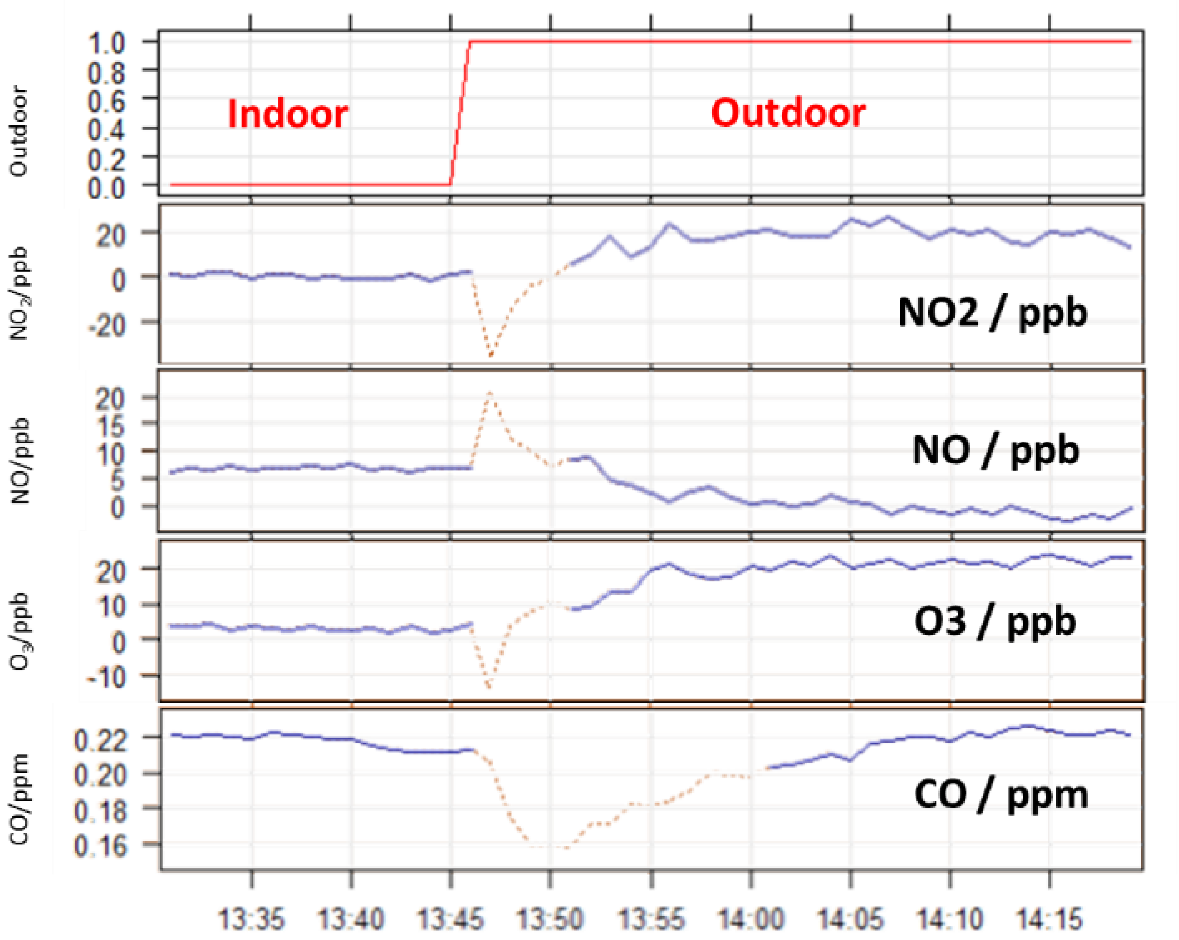

Figure S8. Close-up of one indoor-outdoor transition. The dotted orange line shows the original signal before removing the false temperature response. The blue line shows the remaining measurement after the removal. The stabilisation time after the transition depends on the sensor type with higher times for CO (15 min) and faster recovery times for the rest of the sensors (5 min).
